# Surgical Treatment and Outcomes of Calcaneal Osteomyelitis in Adults: A Systematic Review

**DOI:** 10.7150/jbji.34452

**Published:** 2019-05-21

**Authors:** Marta Sabater-Martos, Irene Katharina Sigmund, Constantinos Loizou, Martin McNally

**Affiliations:** 1 The Bone Infection Unit, Nuffield Orthopaedic Centre. Windmill Rd, Headington. Oxford OX3 7HE, Oxford University Hospitals Foundation NHS Trust, Oxford, UK.; 2 Department of Orthopaedics and Trauma Surgery. Hospital Universitari Germans Trias i Pujol, Badalona. Carretera del Canyet s/n, 08916 Badalona, Spain.; 3 Medical University of Vienna, Department of Orthopaedics and Trauma Surgery, Spitalgasse 23, 1090 Vienna, Austria.

**Keywords:** calcaneal osteomyelitis, systematic review, outcome, treatment, muscle flap, antimicrobial carrier, infection.

## Abstract

**Introduction:** Calcaneal osteomyelitis is an uncommon and challenging condition. In this systematic review we aim to analyse the outcomes from concomitant use of bone debridement and soft tissue management for patients diagnosed with calcaneal osteomyelitis.

**Materials & Methods:** A complete computerised and comprehensive literature search of Pubmed and Cochrane database was undertaken from January 2000 to October 2018. During the review, studies were screened for information about the surgical and antimicrobial treatment, the complications, the reinfection rate and the functional outcome of patients with calcaneal osteomyelitis.

**Results:** Of the 20 eligible studies included, seven (35%) described bone treatment only, six (30%) soft tissue treatment only, five (25%) soft tissue and bone treatment, and two (10%) focused on prognostic factors and differences in outcomes between diabetic and non-diabetic patients.

In the studies with bone treatment only, infection recurrence ranged from 0 to 35% and the amputation rate from 0 to 29%. If soft tissue coverage was also needed, both the reinfection rate and amputation rate ranged from 0 to 24%. Studies presenting the functional status generally showed preservation or even improvement of the preoperative ambulatory status.

**Conclusion:** Calcaneal osteomyelitis is difficult to treat. A multidisciplinary approach involving orthopaedic surgeons, plastic surgeons and infectious disease physicians is preferred. The heterogenicity of studies has hindered the development of agreed treatment protocols, which would be useful in clinical practice.

## Introduction

**Figure 1:**
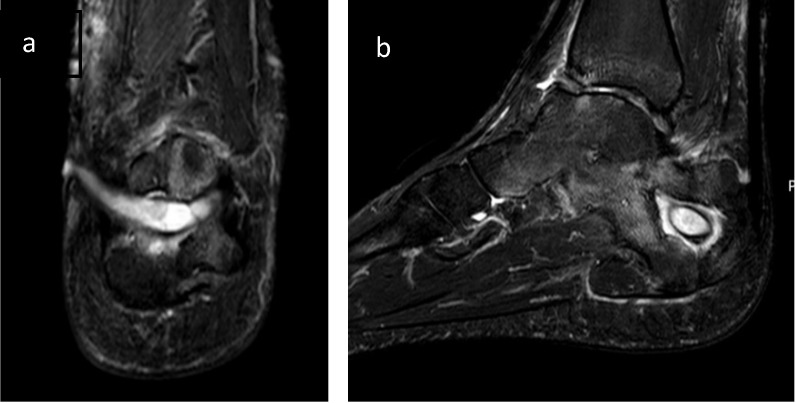
**1a**: Coronal MRI showing central abscess with sinus extending to the medial side. There is no subperiosteal extension of the abscess. This infection followed internal fixation of a closed, complex calcaneal fracture. **1b**: Sagittal MRI showing the classical 'corona' sign of osteomyelitis.

**Figure 2:**
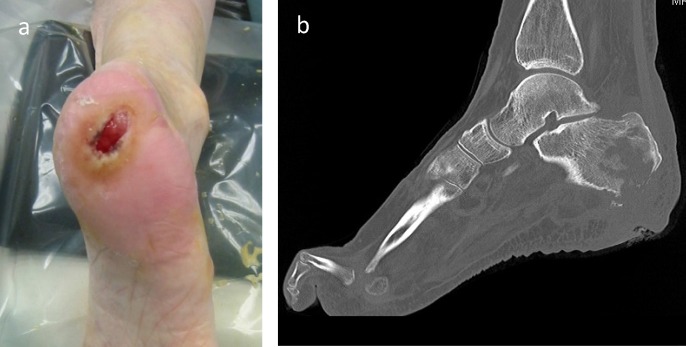
**2a**: Plantar ulceration over calcaneal osteomyelitis in a patient with paraplegia after spinal cord injury. **2b:** CT Scan showing the bone destruction deep to the ulcer.

Calcaneal osteomyelitis poses specific challenges for patients and treating clinicians. Of all osteomyelitis cases, the incidence ranges from 3 to 10% 1 and is often seen in association with diabetes and other comorbidities. Infection most commonly presents after a traumatic event (open fractures or following fixation) or in patients with heel ulceration due to prolonged bed rest or lower limb neuropathy or vasculopathy 1. The diagnosis is primarily based on clinical features (unilateral localized swelling, erythema, localized pain, increased limb temperature, sinus or heel ulceration). Radiological assessment with CT or MRI (Figure 1) and bacteriological analysis can help to confirm the diagnosis 2. The calcaneum is unusual, in that, it has specialised skin attached very tightly to the bone surface with short, dense fibres. Infection within the bone rarely causes sub-periosteal abscesses, but rather erodes through the specialised skin producing ulceration and skin loss 3. It is very difficult to restore this tissue loss (Figure 2). For successful treatment of calcaneal osteomyelitis (infection control and good functional outcome) the following objectives should be delivered:

- excision of all necrotic bone and soft tissue
- good dead space management
- preservation of weight bearing capability
- good soft tissue cover for a well vascularised environment 2.

Nevertheless, treatment options for calcaneal osteomyelitis are limited. Historically, the standard treatment has been lower limb amputation. This treatment is effective in infection eradication but is associated with an inefficient gait 4 as well as reduced physical activity 5 and mortality rates approaching 70% at 5 years in diabetic patients 6. In addition, in patients who cannot apply or remove a prosthesis, can result in them being bed bound or wheelchair bound.

Therefore, appropriate limb salvage procedures for treatment of calcaneal osteomyelitis are of immense importance to achieve infection-free conditions which offer a good functional outcome for these patients. Treatments may involve not only bone debridement techniques such as curettage, partial or total calcanectomy, but also soft tissue management. A great range of treatments are described from primary wound closure or delayed primary wound closure to negative pressure therapies (NPWT), pedicle flaps and microsurgical free flaps 7,8. Nevertheless, evidence on the efficacy and accuracy of combining both treatments are lacking.

The aim of this review was to analyse the concomitant use of bone and soft tissue approaches for patients diagnosed with calcaneal osteomyelitis.

## Materials and method

### Search strategy

**Figure 3:**
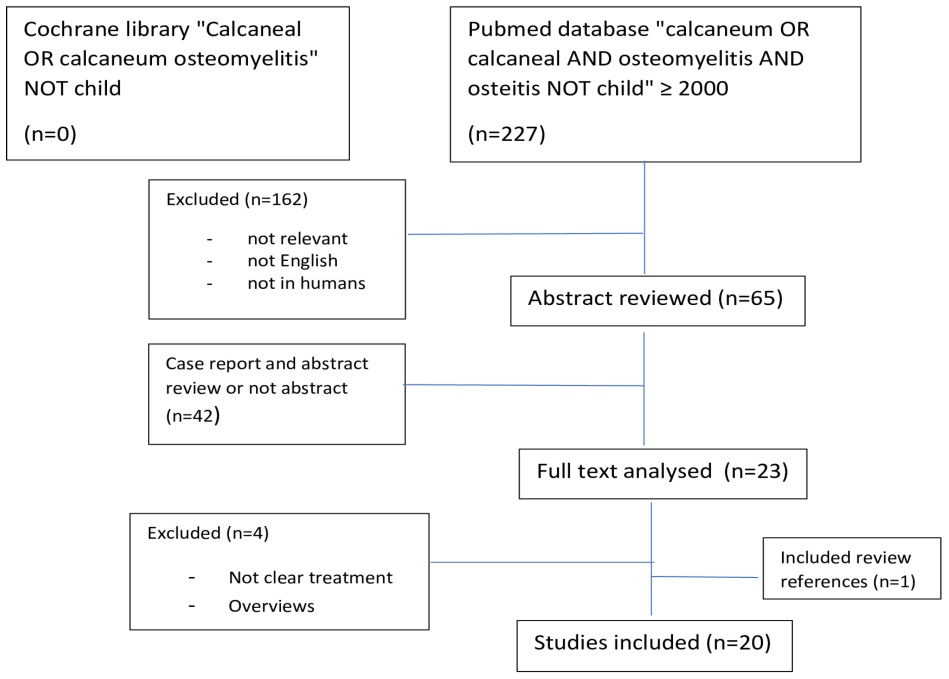
PRISMA flow diagram.

A computerised literature search in Pubmed and the Cochrane library database was conducted. We included articles published in English during the last 19 years, from January 1^st^ 2000 to October 31^st^ 2018. Search terms included “calcaneum”, “calcaneal” “osteomyelitis”, “osteitis”. Calcaneal osteomyelitis in children often has a different aetiology, treatment and prognosis, compared to adult calcaneal osteomyelitis. For this reason, this review does not include cases of calcaneal osteomyelitis during childhood (patients under 16 years old). A full search strategy is shown in figure 3. Any animal or *in vitro* studies were excluded. All levels of evidence except case reports were included. Articles not providing a clear description of the surgical treatment and/or outcome were excluded. Finally, studies not providing an abstract were also excluded. Further studies identified from reference lists were added after review of full papers.

### Study selection

A total of 227 studies were identified from our search. Of these, 162 publications did not meet the inclusion criteria and were eliminated. Full text articles were retrieved for 23 publications. Of these, four were excluded because two did not provide a clear description of the surgical treatment and two were overviews of the condition. Thus, 19 studies met the inclusion criteria and were reviewed in detail. References from these studies were analysed and one further study was included in the review.

### Data extraction

The following data was extracted from all relevant papers: 1) author and journal, 2) year of publication, 3) type of study, 4) number of patients, 5) treatment received, including information on bone resection and debridement, soft tissue and dead space management, antimicrobial therapy and use of adjunctive therapy such as negative pressure wound therapy, growth factors or external fixators, 6) complications, 7) outcome, 8) recurrence of infection and 9) study limitations.

## Results

All the included studies gave a clear description of the surgical treatment received. Of the 20 studies, seven (35%) described bone treatment only, six (30%) focused on soft tissue treatment, five (25%) on both soft tissue and bone treatment and two (10%) described prognostic factors and differences in outcomes between diabetic and non-diabetic patients. All the studies were retrospective except for a prospective study by Paola et al., and a systemic review by Shade, consisting of 16 studies with 100 patients, most treated before 2000. The retrospective studies had an average number of 15.3 patients [range 2 to 42]. Diagnosis of calcaneal osteomyelitis was made either by Magnetic Resonance Imaging (MRI) or confirmed by cultures.

### Bone treatment

A total of 12 studies described the management of infected bone. Of these, five studies also reported the management of soft tissues. Of the seven studies focusing on bone treatment only, five used a specific technique: four studies assessed the outcome after partial calcanectomy and one after the Silo technique. A summary of these studies is shown in Table 1 and Table 2.

#### Partial Calcanectomy

In the study by Bollinger et al. (2002), the 22 included patients showed no recurrences of deep infection 9. However, more than 50% of the patients (n=12) had delayed wound healing, and the poor wound healing rate was even higher (100%) when considering diabetic patients only. Fisher et al. (2010) who assessed a novel approach using the ´Hurricane incision´ in two patients showed no recurrence of infection but a 50% wound breakdown rate 10. This approach consists of a plantar based incision, with 2 semielliptical incisions circumscribing the calcaneal ulceration in order to resect the ulcer in total.

In the study by Oliver et al. (2015), 42 patients with calcaneal osteomyelitis and partial calcanectomy were included 11. The patients were divided into two cohorts with a resection of the calcaneum of <50% and >50%. The lower extremity function and the limb salvage outcome were compared between both groups. They showed that regardless of the resection size, patients have similar function and a similar recurrence rate.

Brooks et al. (2004) reported 17 patients (20 feet) with calcaneal osteomyelitis treated either with partial or total calcanectomy 12. None of the patients had a recurrence of infection. Thirteen patients (76%) were diabetic; nine of those (69%) had surgery previously due to vasculopathy. The healing rate was 65%, while the amputation rate was 30%.

#### Silo Technique

The Silo technique consists of an MRI guided debridement, drilling bone tunnels and injecting antibiotic-loaded bioceramic 13. The study by Drampalos et al. (2017) includes 12 patients, in which either a primary wound closure was done, or a negative pressure wound therapy (NPWT) was used. All the wounds healed, except in one patient where a reverse sural flap was needed at a second stage. None of the patients developed an infection recurrence and only two (17%) patients showed a decreased ambulatory status.

#### Combined Techniques

Babiak et al. (2016) analysed 32 patients for calcaneal osteomyelitis. Of these, eight patients (25%) were treated with a drilling operation combined with insertion of collagen-gentamicin sponges, nine (28%) with debridement with or without fasciocutaneous flap, and 15 (47%) with a partial calcanectomy. Infection control and wound healing was achieved in 75% (n=6/8), 78% (n=7/9), and 73% (n=11/15), respectively 14. Patients treated with bone preservation showed lower infection control rates as well as lower patient satisfaction. However, they had a better walking ability. Failure was treated with total calcanectomy (n=2), amputation (n=4), or a further bone preserving technique (n=2).

In two studies 15,16, Ilizarov circular frames were used to off-load and maintain the foot in equinus position to decrease the plantar soft tissue defect. In the study by Akurtt et al. (2017), they assessed the outcome of 23 patients treated with an Ilizarov frame. The external frame was removed after a mean of 8.2 weeks, decreasing plantar flexion by 1º per day. They reported pin site infections in 69.5% (n=16); in two a removal of the offending wires was necessary. Paola et al. (2016) included 18 patients with a mean external frame time of 11 weeks. The amount of correction per day is not mentioned, nor the pin site infection rate. Both studies agreed on the importance of bone debridement but differ in the technique. The first study used an MRI guided curettage with a 78% of clinical complete cure and 22% partial recovery (3 needed a flap operation and 2 a BKA). The second, advocated partial calcanectomy along with the application of a dermal substitute. They showed a 100% healing rate with no infection recurrence or need of amputation. However, three patients had a wound breakdown.

#### Systematic Review

The last of these seven studies is a systematic review by Schade (2012) including 16 articles (also case reports), mainly published before 2000 comparing the outcome of partial and total calcanectomy 17. The patients after total calcanectomy showed a higher complication rate, amputation rate, and need for ambulatory care in comparison to the patients with partial calcanectomy. Diabetic patients had a five times greater risk of amputation, but variation in the severity of the infection was not fully described.

The other three studies described the management of both bone and soft tissue. All of them were treated with partial calcanectomies, but different variants of soft tissue coverage. For this reason, the results will appear in the next section.

### Soft tissue

A total of 11 studies discuss the soft tissue management for calcaneal osteomyelitis. Five studies reported both soft tissue and bone management (two of them have already been discussed in the bone treatment section), while six are more focused on soft tissue management. A summary of these studies is shown in Table 3 and Table 4.

#### Free Tissue Transfer

Two studies 18,19 assessed the outcome after radical bone debridement and soft tissue reconstruction using a free muscle flap, serratus anterior (n=13) and gracilis muscle (n=3), respectively. The degree or method of bone resection was not described. None of the cases had a recurrence of infection. In one patient, a haematoma occurred below the flap after surgery which was managed conservatively.

#### Local Pedicled Flaps

Al-Qattan (2001) used abductor digiti minimi muscle for soft tissue reconstruction in four patients with calcaneal osteomyelitis. Neither an infection recurrence nor postoperative changes of the neurological status were described 20. Bofelli et al. (2013) reported three cases treated by near total calcanectomy with different rotational flaps depending on the ulceration zone of the heel 21. No infection recurrence or complications were described, and all patients maintained their previous ambulatory status.

In three studies 22-24, patients were treated with aggressive bone and soft tissue debridement followed by reconstruction of the soft tissue defect using neurocutaneous or fasciomusculocutaneous flaps for a well vascularised environment. In all patients (Yildirim et al [n=9; neurocutaneous flap], Chen et al. [n=11; distally based sural fasciomusculocutaneous flap], Wang et al. [n=5; “hybrid” sural flap]), a complete survival of the flaps was demonstrated. In the study by Yildirim et al. (2003) two patients with flap related complications due to venous congestion and partial loss at the donor site were described. Both were managed non-surgically. One patient (11%) continued with persistent pyogenic discharge requiring a partial calcanectomy and a free muscle flap transfer. No study reported a recurrence of infection or amputation.

#### Novel Techniques

Goudie et al. (2012) published a study comparing three different techniques for soft tissue management after partial calcanectomy^25^: split thickness graft (n=5), allogenic bilayered skin substitute (n=2) or recombinant platelet-derived growth factor/oxidised regenerated cellulose/bovine collagen (rhPDGF/ORC/collagen) (n=14). All patients in the first and second technique closure groups showed inadequate wound healing needing the secondary use of rhPDGF. Hence, the use of rhPDGF showed a higher primary healing rate (97%). No recurrence of infection was described. Nonetheless, three patients needed BKA due to reopening of the ulcer within 12 months of healing. Overall, limb salvage rate was 76%.

Two studies 15,26 reported the use of dermal substitute layers without infection recurrence. However, 20% of the patients needed a skin graft or a second dermal layer implantation due to wound healing disorders. The last three studies used NPWT between bone debridement and rhPDGF or dermal layer application.

Negative pressure wound therapy (NPWT) was used in combination with other techniques in 6 studies 12, 13, 15,19, 25, 26. Specific details (duration of use, sponge changes, complications) of NPWT were generally not reported, but Fraccalvieri et al.(2012) used it for 14 days prior to skin grafting. Overall, recurrence rates and amputation rates were not improved compared with those cases without NPWT use.

### Prognostic factors

Two studies specifically investigated risk factors for poor outcome after treatment of calcaneal osteomyelitis. Walsh et al. (2013), showed that even with the same treatment, diabetic patients had a prolonged hospital stay and a lower limb salvage rate compared with non-diabetic patients 27. Merlet et al. (2014) described a 40.5% relapse rate needing multiple and different treatments strategies. Additionally, 33% of the patients presented unfavourable outcome with persistent heel ulceration or amputation 1. Risk factors for poor outcome after surgical treatment of calcaneal osteomyelitis were age >65, ASA Grade >2, diabetes, neuropathy and posttraumatic aetiology.

### Surgical stages

Nine studies described treatment in a single stage 9,10,13,14,16,18,21,23,24. Only 2 papers describe 2 stage procedures 11,20. In both papers, skin closure was performed separately after the first stage bone excision. Finally, 4 papers report a multiple stage technique (≥3 stages) 15,19,25,26.

### Antibiotic

#### Local antibiotic delivery

In the study by Babiak et al. (2016), eight (25%) of 32 patients were treated with collagen-gentamicin sponges (CGS) as a local antimicrobial carrier after debridement and drilling of calcaneum 14. Two (25%) of these patients had a recurrence of infection and were finally treated with BKA, neither being able to walk after amputation. The remaining six (75%) patients had no further surgical treatment for infection recurrence and were able to walk and satisfied with their management.

Drampalos et al. (2017) used in addition to the above mentioned Silo technique an antibiotic carrier consisting of calcium sulphate (CS) and hydroxyapatite (HA) loaded with gentamicin 13. None of the patients showed infection recurrence.

Lastly, Walsh et al. (2013), also used two different localized delivery systems (CS with tobramycin sulphate or gentamicin-collagen haemostat) in all 10 patients included 27. The choice of one system over the other depended on the nature of the wound, previous wound isolates and blood cultures. In their study, diabetic patients performed worse than non-diabetics. Two patients required a BKA after multiple debridements due to infection persistence. Infection recurrence was not properly stated.

#### Antimicrobial therapy after surgery

Twelve studies reported the use of systemic antimicrobial therapy after surgery. All used antibiotics selected according to culture results and antibiogram. However, duration and empiric treatment are not always mentioned. Only nine studies described the specific duration ranging from two to 12 weeks. In three studies 1,22,27, the duration was based on cultures results, wound and soft tissue progression, and blood markers. Limited specific data on microbiology was reported in only four studies 14,15,16,23. Bone cultures demonstrated *Staphylococcus aureus* to be the most common organism involved in calcaneal osteomyelitis.

## Discussion

According to the evidence reviewed over the last 18 years, calcaneal osteomyelitis treatment tried to deliver the following objectives: 1 bone infection control (surgical excision and systemic antimicrobial treatment), 2 dead space and wound management (with or without local antibiotic delivery and wound closure) and 3 a good functional outcome.

Limitations of the included studies are first, the lack of standardized definitions of infection, failure, relapse, recurrence of infection, and wound healing disorders. In some studies, detailed information about important diagnostic protocols, outcome parameters, such as recurrence of infection or functional results, is completely absent. Many studies report no recurrence of infection but list numerous patients with “poor wound healing” or “wound breakdown”. Clearly some of these will have either persistence of the initial infection or recurrence as the cause of the open wound. There is a need for a standardized method of reporting wound problems and careful use of a definition for recurrent infection. Moreover, there is an important potential bias because the extent of the infection is not fully described in all the papers and therefore, may be variable.

Secondly, most of the studies were analysed retrospectively (n=19/20), only one study was conducted prospectively. Another drawback is the small sample size of assessed patients with calcaneal osteomyelitis and the short follow-up. Ideally, all patients should be reviewed for at least one year, but this was only reported in 10 studies. The number of investigated patients ranged from 2 to 100, 80% of the studies included less than 30 patients and 35% less than 10 patients. Due to these limitations, a proper comparison between these studies is difficult and an accurate and reasonable conclusion cannot always be guaranteed. There may be a significant bias in favour of good outcomes with smaller studies reporting no complications.

In this systematic review, 20 studies matching our inclusion criteria were studied in detail. Different surgical approaches related to bone and/or soft tissue management achieved similar reinfection rates and functional outcome. This review has not identified any method which is superior to any other, although partial bone resections may offer better functional outcome compared to total calcanectomy. A study by Brown et al. 28 on diabetic patients with foot ulcers who underwent various levels of amputation such as transtibial, transmetatarsal, Chopart, partial calcanectomy (PC) total calcanectomy (TC) has shown no significant differences in ambulatory status though numbers were small. Interestingly, mortality rates at 5 years for TTA was 45%, for PC was 69%, for TC was 59% and more proximal amputations were required in 5% for TTA 35% for PC and 31% for TCs, demonstrating the poor outcomes whatever the procedure in the diabetic population 28.

Regarding bone management results, bone preserving and more radical procedures seem to have comparable relapse and recurrence rates of osteomyelitis, although functional results may be better with less radical surgeries 11,13,14. However, the functional outcome was not always specified. Only the study by Oliver et al. (2015) assessed the lower limb function objectively by using the Lower Extremity Function Scale (LEFS), in which patients with less aggressive surgeries provided better LEFS functional scores. Other studies assessed the walking capability of their patients and compared it with their preoperative walking ability 9,13,14,17-23,25,26. These studies generally show preservation or improvement of the functional outcome after surgery.

Wound closure can be achieved by various plastic procedures if a primary wound closure is not possible: free muscle flaps (serratus anterior, gracilis), or local flaps (rotational flaps, abductor digiti minimi flap, neurocutaneous or fasciomusculocutaneous flaps). The reviewed studies showed no difference in the reinfection rate and failure rate of the flaps. However, the choice of soft tissue coverage should be based on the location and size of the soft tissue defect 21. Direct closure with the adjacent normal specialized skin is preferable, but small defects may be reliably covered by local pedicle flaps.

No recommendation on the extent of soft tissue defect which can be covered by a local flap can be given from the reviewed literature. In larger defects, free flaps which provide a good obliteration of dead space should be used. They also import well perfused tissue which can deliver antibiotics and immune cells to the area. Disadvantages of free flaps are the need of microsurgery, long operation time, and prolonged hospital stay combined with higher costs. They are also usually insensate, producing a later risk of pressure ulceration. Regardless of which coverage is used, the applied procedure should guarantee an improved bone vascularization and a good dead space management to avoid haematoma formation 8.

NPWT showed no additional benefit in the treatment of calcaneal osteomyelitis. This finding is similar to the experience of NPWT in open fractures 29. A drawback of NPWT is the need for at least one additional surgery for wound closure and often the sponge will need several exchanges, prior to definitive closure 13,15,19,25,26. Furthermore, prolonged use of NPWT can lead to an increased surgical site infection (SSI) rate due to sponge seeding. However, the current literature on this field is controversial and the development of SSI after NPWT depends on wound type and duration of use 29, 30, 31.

The use of external circular frames has shown good results. It seems that this device allows off-loading of the hindfoot and maintains the foot in an equinus position to minimize the soft tissue defect 15,16. However, a standardized treatment algorithm is missing.

The novel use of rhPDGF is an interesting approach which may be helpful in more recalcitrant cases 25.

Only two-thirds of the studies provide a proper description of the antimicrobial therapy used. Generally, an empirical antimicrobial course was given initially after surgery followed by an oral regime based on the microbiological results and antibiogram. For successful infection control, a combined surgical and antimicrobial treatment can be highly recommended and has been well-described in the literature in a wide range of types of bone infection 2, 32, 33. The use of local antibiotic carriers for osteomyelitis shows promising results in the current literature 33. If antibiotic carriers were used in the calcaneum, less radical surgeries were necessary, the osteomyelitis was controlled, and the patients were highly satisfied 13,14,27. However, the numbers reported are small and this modality of antibiotic delivery requires further study.

Calcaneal osteomyelitis can be associated with various comorbidities. Neuropathy and vasculopathy are known to reduce soft tissue healing resulting in higher recurrence and amputation rates 1,27. Merlet et al. (2014), found several prognostic factors for a poorer outcome including neuropathy and diabetes, age >65 years, ASA >2 and posttraumatic origin of the infection. Interestingly, vasculopathy was not reported to be a risk factor for wound healing disorders alone, although diabetes (which often produces vascular insufficiency) was included.

Healing rates after treatment of calcaneal osteomyelitis range from 66% to 100% in all included studies, although every article provided different definitions. Some considered that healing was achieved if there was no need for multiple debridements; others preferred using clinical and/or radiological signs of ongoing infection even if the patient needed repeated debridements or different surgical approaches to control the infection. Studies which investigated diabetic patients generally showed a reduced healing rate.

Overall results favour less radical surgery, with good soft tissue coverage and dead space management. Late recurrences remain challenging, especially in patients with continued neuropathy and new ulceration.

Calcaneal osteomyelitis is difficult to manage and requires a multidisciplinary approach involving orthopaedic surgeons, plastic surgeons and infectious diseases physicians. Studies with standardized treatment algorithms and outcome measures including inputs from all three disciplines would be valuable in clinical practice. However, patients with this condition have varied co-morbidities and different components of disease in the calcaneum, requiring an individual treatment strategy.

## Figures and Tables

**Table 1:** Systematic review of studies describing bone treatment for calcaneal osteomyelitis. Treatment

Reference	Patients (no.)	Diabetes (no.)	Bone treatment	Antibiotic carrier	Soft tissue closure	Postoperative antibiotic
Bollinger et al. 2002	22	9	PC	None	Primary closure	NA
Brooks et al. 2004	17 (20 feet)	13	15 PC and 5 TC	None	Primary closure or NPWT	6 weeks culture specific
Fisher et al. 2010	2	2	PC with Hurricane approach	None	Primary closure	NA
Schade 2012	100 (16 papers)	NA	76 PC and 28 TC	NA	NA	NA
Oliver et al. 2015	42	Cohort 1: 20/25 Cohort 2: 15/17	Cohort 1: <50% PCCohort 2: >50% PC	none	Delayed primary closure	NA
Babiak et al. 2016	32	NA	Bone preserving techniques vs PC and TC	CGS	NA	2 weeks empirical iv or oral + 4 weeks culture specific
Drampalos et al. 2017	12	12	Silo technique	Gentamicin loaded bioceramic	Primary closure or NPWT	Empiric gentamicin + teicoplanin and modified accordingly for 6 to 12 weeks
						
Fraccalvieri et al. 2012	7	2	PC	None	Integra dermal regeneration template + NPWT for 14 days+ skin graft	After both procedures protocol antibiotic, when cultures available a specific antibiotic
Gohds et al. 2012	3	0	Radical debridement with cortical shell preservation	None	NPWT + gracilis free flap	Culture specific antibiotic
Goudie et al. 2012	21	21	PC	None	NPWT +1) split thickness graft, 2) allogenic bilayered skin subsitute or3) rhPDGF/ORC/coll.	NA
Paola et al. 2016	18	1	PC	None	External frame + NPWT + dermal substitute + split thickness skin graft	2 weeks iv followed by 4 weeks oral
Akkurt et al. 2017	23	23	MRI guided debridement	None	External frame	NA

**Table 2:** Systematic review of studies describing bone treatment for calcaneal osteomyelitis. Results and follow up.

Reference	Infection Recurrence	Amputation	Functional outcome	Follow up (months)
Bollinger et al. 2002	0	0	NA	27 (2-80)
Brooks et al. 2004	35%	6 (30%)	NA	NA
Fisher et al. 2010	NA	0	NA	NA
Schade 2012	NA	NA	85% maintain or increase ambulatory status	33
Oliver et al. 2015	NA	Cohort 1: 7 (28%) Cohort 2: 5 (29%)	LEFS Cohort 1: 33.9 Cohort 2: 36.6	Cohort 1: 43Cohort 2: 38
Babiak et al. 2016	Bone preserving: 23.5% PC or TC: 11.8%	4 (12.5%)	Walking abilityBone preserving:100%PC or TC: 93.33%	Minimal of 24 months
Drampalos et al. 2017	0	0	6/8 maintain ambulatory status	4
				
Fraccalvieri et al. 2012	0	0	All patients maintain preoperative mobility	22 (6-36)
Gohds et al. 2012	0	0	All patients return to ambulatory status	NA
Goudie et al. 2012	24%	4 (24%)	57% independent ambulation	24
Paola et al. 2016	0	0	NA	7.5
Akkurt et al. 2017	5 (22%)	2 (8.7%)	78% were able to ambulate	4.5

*Note. NA: not available, Cohort 1(patients who received a <50%PC, cohort 2 (patients who received >50%PC) PC: partial calcanectomy, TC: total calcanectomy, CGS: collagen gentamicin sponge, NPWT: negative pressure wound therapy, rhPDGF/ORC/coll: recombinant platelet-derived growth factor/oxidised regenerated cellulose/bovine collagen, LEFS: lower extremity functional scale Below middle line studies describing both bone and soft tissue treatment

**Table 3:** Systematic review of studies describing soft tissue treatment for calcaneal osteomyelitis. Treatment

Reference	Infection Recurrence	Amputation	Functional outcome	Follow up (months)
Al-Qattan 2001	0	0	NA	36
Yildirim et al. 2003	1 (11%)	0	8/9 returned to presurgical ambulatory status	22 (15- 27)
Chen et al. 2005	0	0	100% returned to presurgical ambulatory status	(6-12)
Xu et al. 2009	0	0	AOFAS score after 6 months: 76	34 (6-72)
Boffeli et al. 2013	0	0	100% returned to presurgical ambulatory status	NA
Wang et al. 2014	0	0	NA	12(6-22)
				
Fraccalvieri et al. 2012	0	0	All patients maintain preoperative mobility	22 (6-36)
Gohds et al. 2012	0	0	All patients return to ambulatory status	NA
Goudie et al. 2012	24%	4 (24%)	57% independent ambulation	24
Paola et al. 2016	0	0	NA	7.5
Akkurt et al. 2017	5 (22%)	2 (8.7%)	78% were able to ambulate	4.5

**Table 4:** Systematic review of studies describing soft tissue treatment for calcaneal osteomyelitis. Results and follow up.

Reference	Patients (no.)	Diabetes (no.)	Bone treatment	Antibiotic carrier	Soft tissue closure	Postoperative antibiotic
Al-Qattan 2001	4	0	Debridement	None	Abductor digiti minimi	NA
Yildirim et al. 2003	9	6	PC with cortical shell preservation	None	Sural or saphenous neurocutaneous flap	3-4 weeks iv followed by 2-3 weeks oral treatment
Chen et al. 2005	11	11	Debridement	None	Sural fasciomusculocutaneous flap	Adjusted according culture results
Xu et al. 2009	13	0	Removal of sequestra and resection od scarred and infected bone	None	Free serratus muscle flap + skin graft	Iv treatment based on culture results
Boffeli et al. 2013	3	0	PC	None	Rotational flap closure	NA
Wang et al. 2014	5	0	Debridement	None	Sural flap with adiponeurofascial portion filling the bone cavity and neurofasciocutaneous portion covering the wound	4 weeks iv + 2 weeks oral treatment adapted to cultures
						
Fraccalvieri et al. 2012	7	2	PC	None	Integra dermal regeneration template + NPWT for 14 days+ skin graft	After both procedures protocol antibiotic, when cultures available a specific antibiotic
Gohds et al. 2012	3	0	Radical debridement with cortical shell preservation	None	NPWT + gracilis free flap	Culture specific antibiotic
Goudie et al. 2012	21	21	PC	None	NPWT +1) split thickness graft, 2) allogenic bilayered skin substitute or3) rhPDGF/ORC/coll.	NA
Paola et al. 2016	18	1	PC	None	External frame + NPWT + dermal substitute + split thickness skin graft	2 weeks iv followed by 4 weeks oral
Akkurt et al. 2017	23	23	MRI guided debridement	None	External frame	NA

*Note. NA: not available, PC: partial calcanectomy, NPWT: negative pressure wound therapy, rhPDGF/ORC/coll: recombinant platelet-derived growth factor/oxidised regenerated cellulose/bovine collagen, AOFAS: American Orthopaedic Foot & Ankle Society Below middle line studies describing both bone and soft tissue treatment
